# Commercially available PGC-1α antibodies vary greatly in specificity and sensitivity

**DOI:** 10.17912/micropub.biology.001268

**Published:** 2024-08-23

**Authors:** Maria Galipeau, Clémence Schmitt, Cindy Baldwin, Aysim Gunes, Jennifer L. Estall

**Affiliations:** 1 Faculty of Medicine, University of Montreal, Montreal, Quebec, Canada; 2 Institut de Recherches Cliniques de Montréal, Montreal, Quebec, Canada

## Abstract

Peroxisome proliferator-activated receptor gamma coactivator 1-alpha (PGC-1α) is an inducible transcriptional coactivator protein involved in mitochondrial biogenesis and metabolism. PGC-1α exhibits a short half-life and low abundance, rendering its detection challenging by immunoblotting. This study compared the specificity and sensitivity of seven commercially available PGC-1α antibodies towards the full-length mouse PGC-1α1 isoform, using overexpression and knockdown in primary mouse hepatocytes. While all antibodies identified overexpressed PGC-1α1, only one detected endogenous PGC-1α1. This study demonstrates wide variability in sensitivity and specificity of PGC-1α antibodies and suggests that controls should be used to differentiate PGC-1α protein from non-specific bands.

**Figure 1. Comparison of PGC-1α antibody sensitivity and specificity for endogenous and overexpressed mouse PGC-1α1 protein by Western blot:**
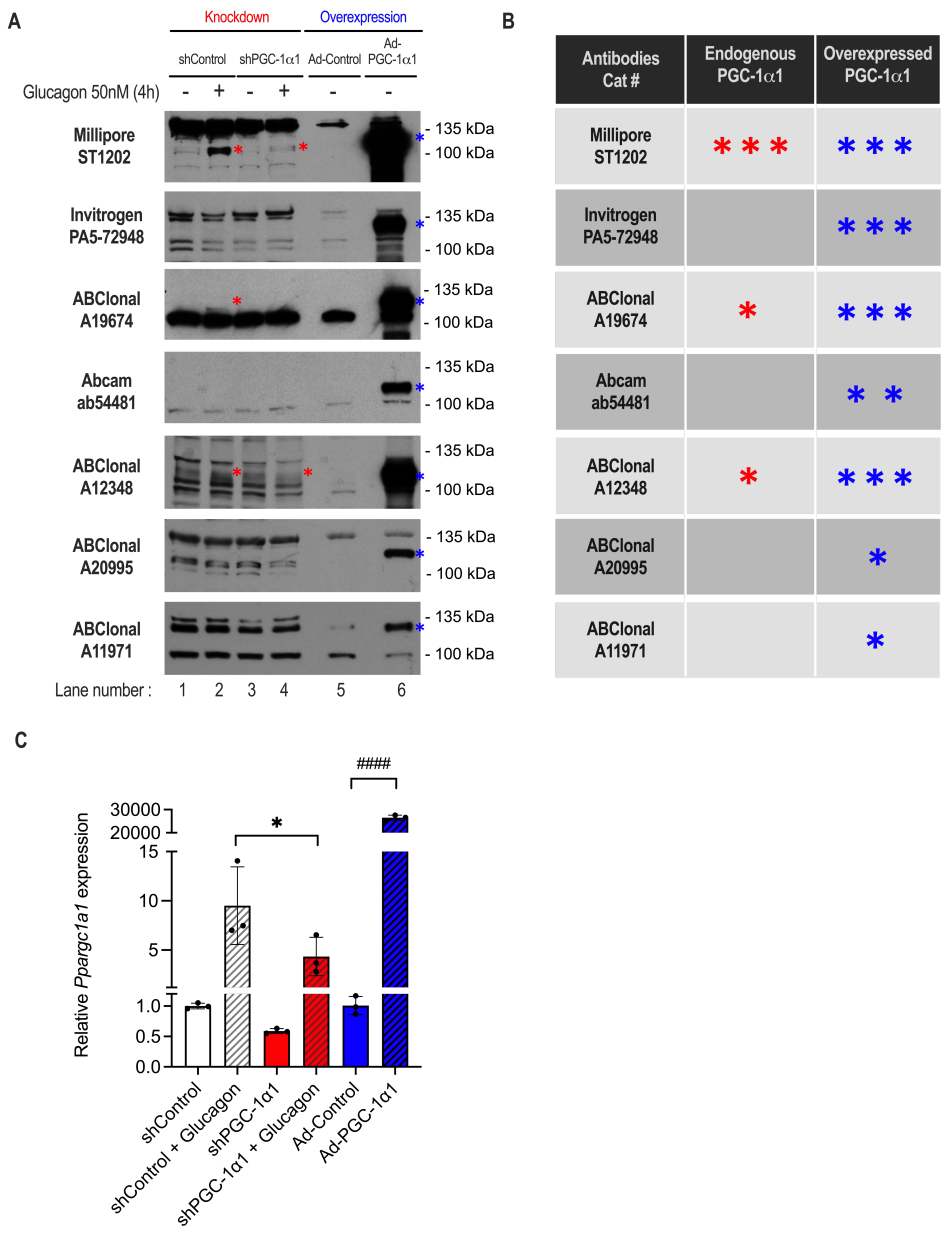
**(A)** Seven antibodies were tested on protein lysates from primary mouse hepatocytes following PGC-1α1 knockdown using shRNA (shPGC-1α1) or overexpression (Ad-PGC-1α1) using adenoviral vectors. Cells expressing shPGC-1α1 or a control shRNA (shControl) were treated with or without 50 nM glucagon for 4 hours to increase endogenous expression. As a positive control, mouse PGC-1α1 was overexpressed (Ad-PGC-1α1) and compared to vector alone (Ad-Control). For each blot, a red star is placed beside the band representing endogenous PGC-1α1 and a blue star beside overexpressed PGC-1α1. Unlabeled bands are either non-specific or other PGC-1α isoforms. **(B)** Comparison table of PGC-1α antibodies. Red and blue stars indicate whether the antibody recognizes endogenous and/or overexpressed PGC-1α1, respectively. Based on qualitative analysis of similarly exposed Western blot films, more stars mean greater sensitivity. **(C)** mRNA transcript levels of mouse *Ppargc1a1* in a separate experiment where primary hepatocytes were treated as in (A). Data are mean±SD of biological replicates from one representative experiment of three independent trials. Statistical analysis was performed using a one-way ANOVA between shControl and shPGC-1α1 or shControl + glucagon and shPGC-1α1 + glucagon [p ≤ 0.05 (*)]. An unpaired t-test was performed between Ad-Control and Ad-PGC-1α1 [p ≤ 0.0001 (####)].

## Description

Peroxisome proliferator-activated receptor gamma coactivator 1-alpha (PGC-1α) regulates genes involved in mitochondrial biogenesis, gluconeogenesis and thermogenesis in response to extracellular stimuli, including fasting, exercise, and cold (Goto et al., 2000; Pilegaard et al., 2003; Puigserver et al., 1998; Rhee et al., 2003; Yoon et al., 2001) . Full-length PGC-1α (also referred to as PGC-1α1 (Ruas et al., 2012) ) is the most well-studied since it was the first to be cloned and functionally described (Puigserver et al., 1998; Vega et al., 2000; Wu et al., 1999) . Multiple antibodies against PGC-1α have been developed and sold commercially for studies of expression, localization, and function in various tissues, cellular contexts and protein applications. However, the specificity and sensitivity of commercially available antibodies targeting PGC-1α are difficult to determine, in part due to multiple non-specific bands at a similar molecular weight that are difficult to identify in the absence of appropriate controls (overexpression and/or knockdown). PGC-1α also has a very short half-life and low abundance in most cell types, largely attributed to its rapid degradation through the ubiquitin-proteasome system (Olson et al., 2008; Sano et al., 2007; Trausch-Azar et al., 2010) , so high sensitivity techniques are needed to detect endogenous levels. Post-translational modifications of PGC-1α can also affect its protein stability, intracellular localization, and molecular weight (Housley et al., 2009; Jäger et al., 2007; Lerin et al., 2006; Puigserver et al., 2001; Rodgers et al., 2005; Rytinki & Palvimo, 2009; Teyssier et al., 2005) . Despite a predicted size of 90 kDa, multiple groups have shown that canonical PGC-1α (PGC-1α1) protein migrates between 110 and 120 kDa on SDS-PAGE gels (Gettys & Chang, 2019; Martínez-Redondo et al., 2016; Ruas et al., 2012; Zhang et al., 2009) . Since PGC-1α1 is often used as a general biomarker of mitochondrial biogenesis, it is important to identify which commercially available antibodies accurately and sensitively identify changing levels of PGC-1α1.

The purpose of this study was to evaluate the performance of commercially available PGC-1α antibodies. Primary hepatocytes were chosen as a model because they express canonical PGC-1α1 protein (Puigserver et al., 2003; Yoon et al., 2001) , yet have a moderate abundance compared to other cell types (i.e. oxidative muscle). To identify endogenous PGC-1α1 protein, PGC-1α1 was knocked down or overexpressed using adenoviral vectors expressing an shRNA (shPGC-1α1) or PGC-1α1 cDNA (Ad-PGC-1α1) respectively, alongside their appropriate control empty vectors (shControl or Ad-Control). As basal hepatic PGC-1α1 protein levels are low, shRNA-transduced hepatocytes were stimulated with glucagon as a fasting signal to induce PGC-1α1 expression (Lin et al., 2002, 2003; Yoon et al., 2001) . We then performed Western blot analysis for mouse PGC-1α1 using seven distinct antibodies and compared their relative abilities to detect both endogenous and overexpressed PGC-1α1 ( Figure 1A,B ). qPCR detecting canonical exons 1 and 2 of PGC-1α was also performed, illustrating the relative extent of knockdown and overexpression of *Ppargc1a1* transcript levels in our model system ( Figure 1C ).

Our findings revealed that all the tested antibodies could detect overexpressed mouse PGC-1α1 (Ad-PGC-1α1), as shown by an intense band between 100 and 135 kDa, compared to vector alone lysates ( Figure 1A, lane 5 vs 6), albeit with varying sensitivity levels. Based on similar exposure times and antibody dilutions, the Millipore ST1202, Invitrogen PA5-72948, ABClonal A19674 and A12348 antibodies exhibited the highest sensitivity towards overexpressed PGC-1α1 ( Figure 1A,B ), while the least sensitive antibodies were ABClonal A20995 and A11971.

Using the positive control to identify the PGC-1α1 migration profile, we evaluated which antibodies were able to detect endogenous PGC-1α1 protein ( Figure 1A, lanes 1-4). All PGC-1α1 antibodies produced bands at various heights. However, when comparing the intensity of these bands across vehicle, glucagon-treated or knocked down conditions, only three antibodies showed the diminished intensity of a band around 110-120 kDa expected following knockdown by shPGC-1α1. Millipore ST1202, ABClonal A19674 and A12348 antibodies detected the glucagon-induced increases in endogenous PGC-1α1 ( Figure 1A, lane 1 vs 2, marked by a red star) which were also reduced by shPGC-1α1 ( Figure 1A, lane 3 vs 4). This suggests that the remaining signals on the Western blots were either non-specific bands or representative of other PGC-1α isoforms. Thus, in our model system, Invitrogen PA5-72948, Abcam ab54481, ABClonal A20995, and ABClonal A11971 antibodies failed to detect endogenous PGC-1α1, summarized in Figure 1B . It is noteworthy that for ABclonal A19674, the faint endogenous PGC-1α1 band was greatly obscured by a closely associated non-specific band ( Figure 1A, lane 2, marked by a red star). Overall, inclusion of appropriate controls was crucial to differentiate endogenous PGC-1α1 from non-specific bands with all commercial antibodies.

For accurate detection of the endogenous mouse PGC-1α1 isoform, we recommend inducing PGC-1α expression using stimuli (such as glucagon in liver) or an expression vector as a positive control for estimation of antibody sensitivity, relative expression levels and migration pattern in all biological contexts. The use of a negative control (knockdown or knockout) is extremely useful to determine specificity of the chosen PGC-1α1 antibody. Our findings suggest that the Millipore ST1202 antibody has both high specificity and sensitivity for PGC-1α1 protein.

In conclusion, this study underscores the importance of validating PGC-1α antibodies. Four of the seven commercially available PGC-1α antibodies tested lacked the sensitivity to detect endogenous PGC-1α1 in primary mouse hepatocytes, whereas all could detect vastly overexpressed levels, but to highly varying degrees. Given the potential for cell-type and species-specific variability in results, it is essential to validate PGC-1α antibodies using appropriate controls.

## Methods

**Hepatocyte cell isolation and culture**

Primary hepatocytes from 2–3 chow-fed (Teklad Global 18% Protein Rodent Diet, TD.01432) C57/B6N male mice (aged 10–12 weeks) were isolated using collagenase (Liberase, Roche) perfusion and Percoll gradient purification. Cells were plated at a density of 450,000/well in 6-well plates in DMEM (Wisent, #319-005-CL) Plating Medium containing 10% FBS (Wisent, #090-150), 2 mM sodium pyruvate (Wisent, #600-110-EL), 1 µM dexamethasone (Sigma D4902), 1% penicillin/streptomycin (Wisent, #450-201-EL), and 0.1 µM insulin (Sigma I-6634). After 2 hours, medium was exchanged with DMEM Maintenance Medium supplemented with 0.2% fat-free BSA (GenDEPOT, #A0100-010), 2 mM sodium pyruvate, 1% penicillin/streptomycin, 100 nM dexamethasone, and 1 nM insulin. The next day, primary hepatocytes were infected overnight with different adenoviral constructs in Maintenance Medium. Media was renewed 24 hours later.

**Hepatocyte cell transduction**

To knockdown mouse PGC-1α1, primary hepatocytes were transduced overnight with a silencing adenovirus pAdTrack-U6-shPGC-1α1 or shControl vector expressing a hairpin that does not recognize any known mouse or human mRNA. The vector expresses a GFP reporter transcribed by a separate CMV promoter. 48 hours post-transduction, primary hepatocytes were incubated overnight in DMEM medium containing 0.2% fat-free BSA, 2 mM sodium pyruvate, and 1% penicillin/streptomycin and stimulated with 50 nM of glucagon or vehicle (PBS) for 4 hours prior to cell lysis.

To overexpress mouse PGC-1α1, primary hepatocytes were transduced overnight in Maintenance Medium with an adenoviral vector pAdTrack-CMV expressing mouse PGC-1α1 or empty vector (expressing a GFP reporter transcribed by a separate CMV promoter). After 16 hours, the media was renewed and cells were incubated up to 48 hours.

**Western blotting**

Primary hepatocytes were lysed on ice in 100 µL RIPA buffer (150 mM sodium chloride, 1% NP-40, 0.5% sodium deoxycholate, 0.1% SDS, 50 mM Tris, pH 8.0) with protease and phosphatase inhibitors (ThermoFisher, Pierce Protease (A32963) and Phosphatase (A32957) Inhibitor Tablets). Total protein was quantified by DC protein assay (Bio-Rad, #5000113-5000114) as per the manufacturer’s instructions. Protein was denatured in 5x Laemmli sample buffer (10% sodium dodecyl sulfate, 25% 2-mercaptoethanol, 30% glycerol, 0.05% bromophenol blue, 292 mM Tris HCl pH 6.8) at 95°C for ten minutes before loading onto a 7.5% - 10% gradient acrylamide gel and electrophoresing at 200V in 1x running buffer (25 mM Tris, 190 mM glycine, 0,1% SDS). 40 µg of protein was loaded to visualize endogenous PGC-1α1 and 20 µg of protein was used for samples with overexpressed PGC-1α1. Protein was transferred onto a nitrocellulose membrane at 25 volts for 20 minutes in 1x transfer buffer (Biorad, #10026938) using the Trans-Blot® Turbo™ Transfer System (Biorad, #1704150EDU). Membranes were blocked for one hour at room temperature in TBS with 5% skim milk powder (20 mM Tris, 130 mM NaCl, 2.5mM EDTA pH 7.6), and incubated overnight in primary antibody (anti-PGC-1α, see table 1) diluted 1:1000 as per manufacturer’s instructions in TBS-0.1% Tween, 5% BSA (or TBS-0.1% Tween, 2% milk for Millipore ST1202 antibody) and incubated at 4°C with continuous rocking. Following 3 x 10 minutes washes in TBS-0.1% Tween at room temperature, membranes were incubated in secondary antibody conjugated with HRP in TBS-0.1% Tween, 5% milk for one hour at room temperature, washed 3 x 10 minutes in TBS-0.1% Tween and visualized using Clarity Western ECL Prime (Biorad, #1705061) reagents. Protein signals were revealed after 1 hour of Blu-Lite UHC™ film exposition (Ultident, #39-20810).

**RNA extraction and qRT-PCR**

Total RNA was extracted from primary hepatocytes using TRIzol reagent (Invitrogen, #15596018) as indicated by the manufacturer’s protocol. RNA concentration and purity were determined by Nanodrop 2000 spectrophotometer (Thermo Scientific). DNAse-treated RNA (1 µg) was reversed transcribed (High-Capacity cDNA Reverse Transcription Kit; Applied Biosystems) and RT-qPCR performed (G892, BlasTaq qPCR MasterMix; Applied Biological Materials) in a 20 µL reaction according to the manufacturer's protocol. The primer sequences used were as follows: *Ppargc1a1* (forward primer, 5'-GGA CAT GTG CAG CCA AGA CTC T-3'; reverse, 5’-CAC TTC AAT CCA CCC AGA AAG CT -3’) and *Hprt* (forward primer, 5'-GGC CAG ACT TTG TTG GAT TTG-3'; reverse, 5’- TGC GCT CAT CTT AGG CTT TGT-3’). Relative gene expression levels were calculated using the ΔΔC _t_ method and normalized to *Hprt* . All reactions were performed in triplicate, and data were analyzed using GraphPad Prism.

## Reagents

We are happy to provide any vectors upon request.

**Table:** 

**Adenoviral plasmids and species**	**shRNA sequences**
pAdTrack-U6-shControl	CAACAGCCACAACGTCTATA
pAdTrack-U6-shPGC-1α1 ( *mus musculus* )	ACTCTGTATGGAGTGACATA

**Table:** 

**Adenoviral plasmids and species**	**Company & catalog number**
pAdTrack-CMV-Control vector	Addgene # 16405
pAdTrack-CMV-PGC-1α1 ( *mus musculus* )	Addgene # 14426

**Table:** 

**Source & catalog number**	**Dilution**	**Animal and clonality**	**Immunogen**	**Species reactivity**	**Tested WB applications by the company**
Millipore, ST1202	1:1000, TBS-0.1% Tween, 2% milk	Mouse Monoclonal	A recombinant protein corresponding to amino acids 1-120 of mouse PGC-1α	Mouse, human and rat	Tested in mouse, human and rat
Invitrogen, PA5-72948	1:1000 in TBS- 0.1% Tween, 5% BSA	Rabbit Polyclonal	A synthetic peptide corresponding to amino acids 400-550 of human PGC-1α protein (#Q9UBK2)	Hamster, human, and rat, but published in human, mouse, and rat	Tested in human and mouse cell lines: A-431, HeLa, HepG2, NIH/3T3, as well as in human adipose and skeletal muscle
Abcam, ab54481	1:1000 in TBS- 0.1% Tween, 5% BSA	Rabbit Polyclonal	A synthetic peptide corresponding to amino acids 750-798 of human PGC-1α (#Q9UBK2)	Human and mouse	Tested in human and mouse
ABClonal, A19674	1:1000 in TBS- 0.1% Tween, 5% BSA	Rabbit Monoclonal	A synthetic peptide corresponding to amino acids 699-798 of human PGC-1α/β (NP_037393.1)	Mouse and rat	Tested in mouse liver and rat heart
ABClonal, A12348	1:1000 in TBS- 0.1% Tween, 5% BSA	Rabbit Polyclonal	A recombinant fusion protein corresponding to amino acids 610-798 of human PGC-1α (NP_037393.1, #Q9UBK2)	Human, mouse and rat	Tested in 293T cells, mouse skeletal muscle, mouse kidney and rat kidney
ABClonal, A20995	1:1000 in TBS- 0.1% Tween, 5% BSA	Rabbit Monoclonal	A recombinant fusion protein corresponding to amino acids 340-480 of human PGC-1α (NP_037393.1, #Q9UBK2)	Human, mouse and rat	Tested in HeLa cells, mouse kidney, rat kidney and rat stomach
ABClonal, A11971	1:1000 in TBS- 0.1% Tween, 5% BSA	Rabbit Polyclonal	A recombinant fusion protein corresponding to amino acids 610-710 of human PGC-1α (NP_037393.1, #Q9UBK2)	Human, mouse and rat	Tested in mouse kidney, mouse heart and rat heart
